# Magnitude of flat foot and its associated factors among school-aged children in Southern Ethiopia: an institution-based cross-sectional study

**DOI:** 10.1186/s12891-023-07082-6

**Published:** 2023-12-13

**Authors:** Asaminew Birhanu, Khaleel Nagarchi, Firdawek Getahun, Mathewos Alemu Gebremichael, Habtamu Wondmagegn

**Affiliations:** 1 Department of Anatomy, College of Medicine and Health Sciences, Arba Minch University, Arba Minch, Ethiopia; 2 Department of Public Health, College of Medicine and Health Sciences, Arba Minch University, Arba Minch, Ethiopia; 3 Department of Public Health, College of Health Sciences, Bonga University, Bonga, Ethiopia

**Keywords:** Flat foot, Pes planus, Magnitude, Gamo Zone, Ethiopia

## Abstract

**Background:**

Flat foot, also known as pes planus, is a common condition among primary school children and is a leading cause of all clinical visits related to foot problems worldwide. It can cause skeletal problems and joint misalignment. This study aimed to assess the magnitude of flat foot and its associated factors among public primary school children.

**Methods:**

An institutional-based cross-sectional study was conducted on 1072 school children aged 11 to 18 years. A structured questionnaire was used for data collection and the footprints were used to calculate the plantar arch index. Data were entered into Epi data version 4.6, and analyzed by STATA version 15. Bivariable and multivariable binary logistic regressions were conducted. Adjusted odds ratios (AORs) with corresponding 95% confidence intervals (CIs) were calculated. Statistical significance was declared at a *P*-value < 0.05.

**Result:**

Out of 1022 participants, 105(10.27%) 95%CI: 8.5–12) had a flat foot. Being male (AOR = 2; 95%CI:1.22-3.30), living in highland altitude (AOR = 8.83; 95% CI: 4.64-16.79), living in midland altitude (AOR = 3.32;95% CI:1.75-6.29), living in an urban area (AOR = 2.42;95% CI:1.15-5.09), insufficient physical activity (AOR = 8.78;95% CI: 4.42-12.3), wearing closed-toe shoes (AOR = 2.33;95%CI:1.27-4.28), obesity (AOR = 6.30;95% CI:3.31-11.9), and foot pain (AOR = 3.52;95%CI:2.08-6.27) had a higher likelihood of flat foot as compared to their counterparts.

**Conclusion:**

One in every ten children had a flat foot. Altitude, residence, sex, physical activity, foot pain, body mass index, and type of footwear were found to be factors statistically associated with flat foot. Integrated interventions for children to have sufficient physical activity, wearing sandals, maintaining a healthy body mass index, and flatfoot screening and monitoring are recommended.

## Background

The foot acts as the foundation for the erect human body – its intrinsic structure and positioning can either directly or indirectly influence all joints of the lower limb, pelvis, and spine [1]. There are 26 bones, 33 joints, and more than 100 ligaments, muscles, and tendons in each foot that act together to transmit force between the lower limb and the ground, allowing stable locomotion and stance [1]. The support of the body weight in the erect posture involves both counterbalancing of the gravitational load, and dynamic equilibrium maintenance [1, 2]. The two known and important arches of the normal foot are the longitudinal and transverse arches. The longitudinal arch of the foot is further subdivided into a medial (MLA) and a lateral longitudinal (LLA) arch [2]. This arch affords an elastic connection between the forefoot and hindfoot [3, 4].

Flat foot describes a condition in which the longitudinal and/or medial arches of the foot are dropped down or flat, and the entire bottom of the barefoot is in contact with the floor or ground surface during standing, walking, or eight-bearing activity [5]. Flatfoot can be due to either inherited or acquired conditions with both flexible and rigid subtypes. Flexible flatfoot is characterized by a normal arch when not bearing weight, but a lowered arch when weight is applied. Rigid flatfoot, on the other hand, is characterized by a significantly reduced range of motion when not bearing weight and a lowered arch in all positions. It is more commonly associated with underlying pathology, which should be thoroughly investigated [6]. Both children and adults can be affected by flat-footedness. However, it is more common in children [7]. Children’s growing structures are more subjected to changes. MLA formation may extend up to the end of the first decade and can be considered permanent, and long-term use of orthotics will be required to prevent future defects in the feet, lower extremities, and spine [8].

As feet are the foundation for the body, a flat foot alters the biomechanical center of gravity and motor chain of the body, this increases stress on the ankle, knee, hip, and spine joints causing gait and postural defects in all age groups [8]. People with flat foot are at risk of chronic foot and knee pain, foot injuries, stress fractures, ankle arthritis, plantar fasciitis, and poor exercise performance [9]. Flat foot is also significantly associated with some developmental delays in children such as delayed walking [10]. Children with flat foot complained of frequent foot pain and difficulty while walking long and fast, running, maintaining balance, and walking on uneven ground. They are usually concerned about the appearance of their feet. They have difficulty in finding suitable footwear, an increased risk of falls, and other deformities in their future life, such as scoliosis and posture problems, as well as a reduced quality of life [11, 12]. These factors have a significant influence on daily activities which demands appropriate interventions. There is no confirmed supportive evidence that osseous misaligned structures will autocorrect over time [10]. Unless treated, misaligned feet do not get better, and they are usually progressively debilitating. Failure to address this disease's progress early will lead to deleterious effects on many parts of the body, including physical and mental health [10].

Flat foot is a common problem among primary school children and it must be addressed by responsible organizations. It is estimated that approximately 20% to 37% of the population in the world has flat foot [9]. Of all clinical visits related to foot problems worldwide, about 90% are due to flat foot [11]. The overall prevalence of flat foot in the city of Gondar in northwestern Ethiopia was 17.6% among children aged 11 to 15 years. This also means that more than one in six children between the ages of 11 and 15 suffers from flat foot. Consequently, flat foot is a public health problem in Ethiopia and Worldwide [12]. Several studies have shown that the magnitude of flat foot and associated factors vary among societies and geographical locations [3, 5, 13–15]. These calls for localized studies that intend to identify area and population-specific factors associated with the magnitude of flat foot. Furthermore, to our knowledge, it has not yet been assessed in the Gamo zone. The findings of the present study will help to enhance the awareness of healthcare providers, policymakers, and other concerned stakeholders to design appropriate evidence-based interventions, early detection, prevention, and rehabilitative strategies for the problem of flat foot and its complications.

## Methods and materials

### Study area and period

The study was conducted in the Gamo Zone in southern Ethiopia from June 15 to August 30, 2021. Arba Minch town is the capital city of the Gamo zone and is found at an elevation of 1285 m above sea level. The zone area covers approximately 47% highland AEZ (agro-ecology zone) which is above 2400 m.a.s.l (meters above sea level); 27% is within the range of 1800-2400 m.a.s.l. was the midland AEZ and 26% is below 1800 m.a.s.l. was the lowland AEZ [16]. There are eighteen woredas in the Gamo zone. Among these, six are located in highland areas, while the remaining seven and five woredas are in midland and lowland areas, respectively.

According to the education authority office of the Gamo Zone, there were about 454 primary schools and 105,595 school children were attending primary schools in Gamo Zone in the 2021 academic year. According to the Ethiopian Demographic and Health Survey (EDHS, 2016), the primary school net attendance ratio (NAR) is 71% (72% girls and 71% boys).

### Study design

An institutional-based cross-sectional study was conducted.

#### Population

All primary school children aged 11 to 18 years in the Gamo Zone were a source population. All primary school children aged 11 to 18 years who were able to ambulate independently and attend selected primary schools in the Gamo Zone were the study population. However, children with a history of lower limb fracture, dislocation in the legs or, limb surgery, and congenital anomalies in the lower limb were excluded from the study.

#### Sample size determination

The sample size was calculated by the double population proportion formula by using OpenEpi version 3. Important factors such as age, sex, body mass index, footwear type, and physical activity were considered [3, 13, 17–19]. The sample size was determined based on the following assumptions: 95% confidence level, power of 80%, a ratio of unexposed to exposed (1:1), design effect 1.5, and by adding 10% nonresponse rates. The maximum sample size was obtained from the variable age (13 years versus 15 years). By considering 15 years as the unexposed group, with a percent of unexposed with outcome (9.9%), and odds ratio (OR = 1.97), we obtained a total of 1072 as a final sample size for the present study.

### Sampling technique and procedure

**Fig. 1.**
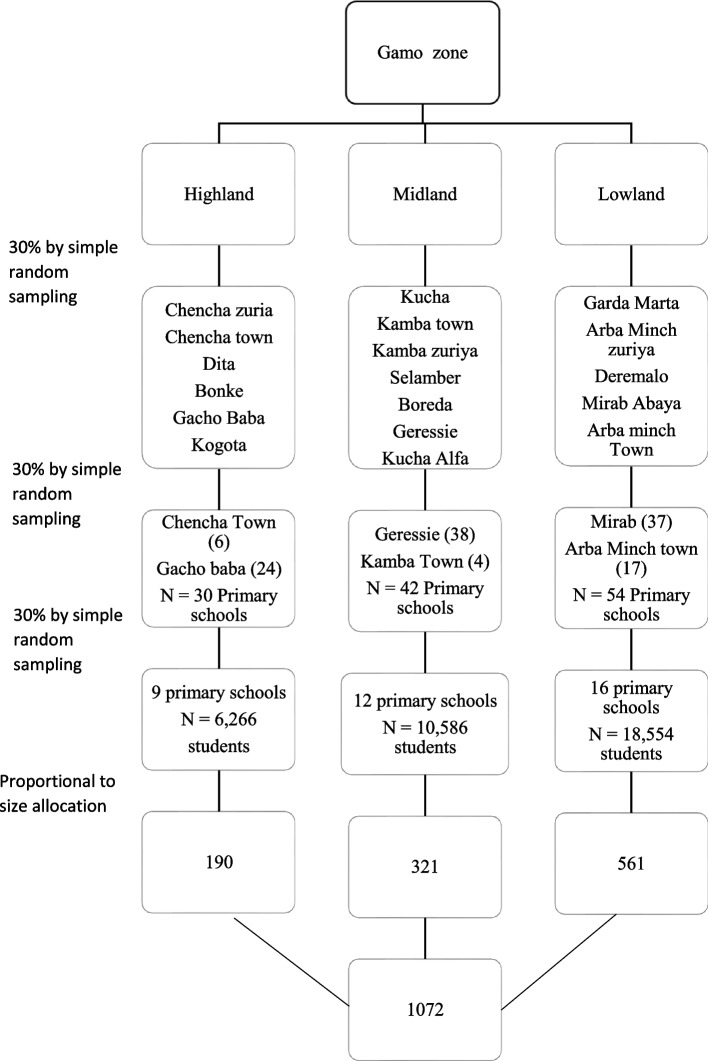
Schematic representation of the sampling procedure on magnitude of flat foot and its associated factors among primary school children in Gamo Zone, southern Ethiopia, 2021

A stratified multistage sampling technique was used. Gamo Zone Woredas were stratified into lowland, midland, and highland areas. Then, 6 (30%) Woredas were selected from each group by the lottery method. Among 126 primary schools in selected woredas, 37 primary schools were selected by the computer-generated random number method (9 schools in highland areas, 12 from midland areas, and 16 from lowland areas). Based on the number of students, the total sample size was proportionally allocated and then the samples were selected by using a simple random sampling technique for which the name register was used as a sampling frame (Fig. 1). Schematic representation of the sampling procedure on magnitude of flat foot and its associated factors among primary school children in Gamo Zone, southern Ethiopia, 2021.

## Study variables

### Dependent variable

Flat foot.

### Independent variable

Socio-demographic variables: age, sex, residence.

Foot wearing related factors: wearing shoes, type of shoes, age of the footwear.

Anthropometric factors: height, body mass index, foot size.

Geographic factor: Altitude.

Clinical factor: foot pain.

Physical activity.

### Operational definition

Flat foot was defined when Staheli’s plantar arch index value of an individual’s one or both feet was > 1.15 [20].

Symptomatic flat foot: Participants who self-reported foot pain in the past 6 months that was not related to any known disease and were diagnosed by Staheli’s plantar arch index as a flat foot [13].

Physical activity:-if a child had < 180 min per week. He or she was categorized as having insufficient physical activity and >  = 180 min per week. He or she was categorized under a sufficient physical activity group [13].

The body mass index for age and sex (BMI) in kg/m^2^ was computed to determine the nutritional status of the children. It was classified depending on Z score value; underweight < -2SD, normal -2SD to1.99SD, overweight 1SD to 2SD, and obese > 2SD [21].

Altitude was categorized as highland which is above 2400 m.a.s.l, midland was within the range of 1800-2400 m.a.s.l. and lowland was below 1800 m.a.s.l [16].

The type of shoes or foot wear was categorized as a close-toe shoe which stands for shoes that cover the entire foot including the top of the foot and heels, and Sandals which stands for the bottom part held onto the foot by straps [13].

### Data collection tool

The data collection tool was developed by reviewing different studies [3, 12, 14, 16, 22]. The questionnaire had six parts. These were socio-demographic characteristics, anthropometric variables, footwear-related factors, geographic factors, clinical factors, and physical activity. The footprints were collected using two smooth wooden plates, normal wall paint, a brush, and a sheet of paper on which the questionnaire was printed. The height of the individuals (in cm) was measured with a stadiometer and their weight (kg) was obtained using a digital weighing scale without shoes and a school bag.

### Data collection procedures

The feet were first cleaned thoroughly. The participant was placed in a sitting position and then asked to dip the foot to be studied into a tray filled with ink. The foot was removed from the tray and the participant was asked to stand up to print the foot firmly on a sheet of paper attached to a wooden platform while flexing the ipsilateral knee slightly (up to 30°). Each footprint was obtained in the standing position with the limb bearing about 50% of the body weight. The above procedures were repeated for the contralateral foot.

**Fig. 2:**
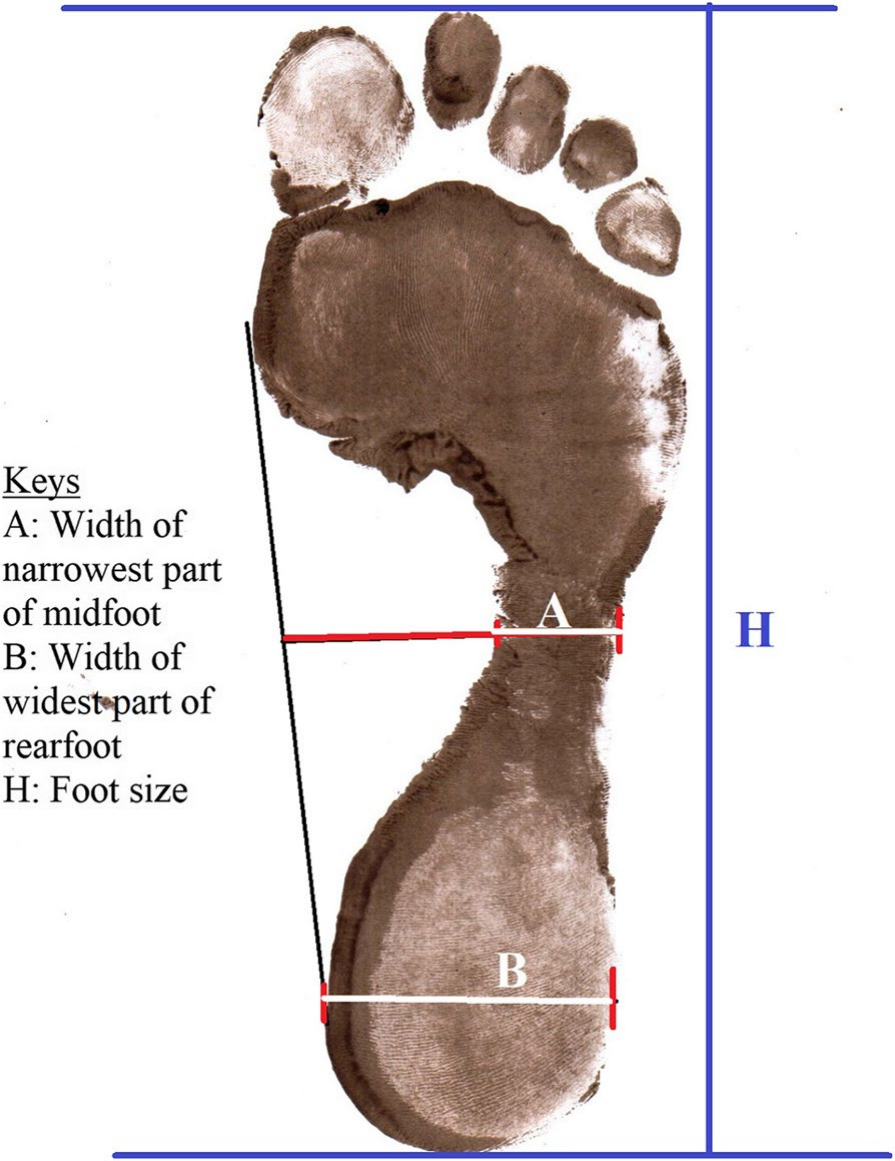
The image of the footprint on plain paper to analyze and determine the PI

The footprints were then used to calculate the plantar arch index (PI). The footprint parameter is better than other parameters because it is inexpensive, easy to handle, and effective for individual and population-based investigations [22]. Using a lead pencil, a line was drawn tangent to the medial midfoot edge and the heel region. The midpoint of this line was determined. From this point, a perpendicular line was drawn crossing the footprint. The same procedure was repeated for the heel tangency point. The perpendicular distance (A; the perpendicular line representing the width covered by the ink from the medial edge to the lateral edge of the midfoot) was measured. Additionally, a second perpendicular distance (B; the perpendicular line representing the width covered by the ink from the medial edge to the lateral edge of the rear foot) was measured. The PI was calculated by dividing the value of A by the value of B. An individual was considered to have a flat foot if his/her SPAI (Staheli’s plantar arch index) value was > 1.15 [15]. SPAI is a well-validated method of measuring the PI [17] (Fig. 2), the image of the footprint on plain paper to analyze and determine the PI.

### Data processing

The collected data were checked for completeness and consistency, coded, entered into Epi data version 4.6, and then exported to STATA version 15 for further management and analysis.

### Statistical analysis

Descriptive statistics such as frequency, proportion, mean, and standard deviation were computed to determine the characteristics of the study subjects. In addition, numeric variables were further explored for normality by using the Shapiro–Wilk test. Therefore, the distributions of weight, width of the narrowest part of the midfoot, width of the widest part of the hindfoot, and SPAI were not normal, hence medians with IQRs (interquartile ranges) were used. To identify the associated factors, a binary logistic regression model was applied. Both bivariable and multivariable binary logistic regression were fitted. Independent variables with a *P*-value ≤ 0.25 in the bivariable regression analysis were entered into multivariable regression. To assess the strength of association, AORs with 95% CIs were computed. A significant association was declared at a *P*-value < 0.05. The presence of multicollinearity in the model was evaluated through the use of variance inflation factor (VIF). The results revealed that the mean and maximum VIF of variables incorporated in the model were 2.30 and 5.31, respectively. Additionally, the model’s fitness was evaluated by administering Hosmer and Lemeshow's goodness of fit test, which demonstrated a strong fit with a *p*-value of 0.41. The mode was a good fit, and no multicollinearity was detected. Pearson’s Correlation Coefficient was used to determine the strength of association between the SPAI with height and Foot size of children. An independent sample t-test was conducted to see the mean difference in SPAI between sexes.

## Results

### Socio-demographic characteristics of the respondents

A total of 1022 children were included in the study yielding a response rate of 95.3% (4.7% of students were not included in the study due to their absence from school and unwillingness to participate). Among these, 568 (55.58%) were females. The majority of participants, 791 (77.40%) resided in urban areas. The mean age of respondents was 13.8 ± 0.062 (± SD (standard deviation)) years. Concerning the altitude of respondents’ lives, 546(53.42%) children were from low-altitude areas, 297(29.06%) were from midland areas and 179(17.51%) were from high-altitude areas (Table 1).

**Table 1:** Socio-demographic characteristics of school-aged children in Gamo zone, Southern Ethiopia, 2021

Characteristics (*n* = 1022)	Categories	Frequency (n)	Percent (%)
Age (in years)	11–14	688	67.32
	15–18	334	32.68
Sex	Female	576	56.36
	Male	446	43.64
Residence	Rural	231	22.60
	Urban	791	77.40
Woreda	Arba Minch Town	494	48.34
	Chencha	122	11.94
	Gacho Baba	57	5.58
	Geressie	139	13.60
	Kamba	158	15.46
	Mirab Abaya	52	5.09
Altitude	Lowland	546	53.42
	Midland	297	29.06
	Highland	179	17.51

### Anthropometry characteristics of participants

**Fig. 3:**
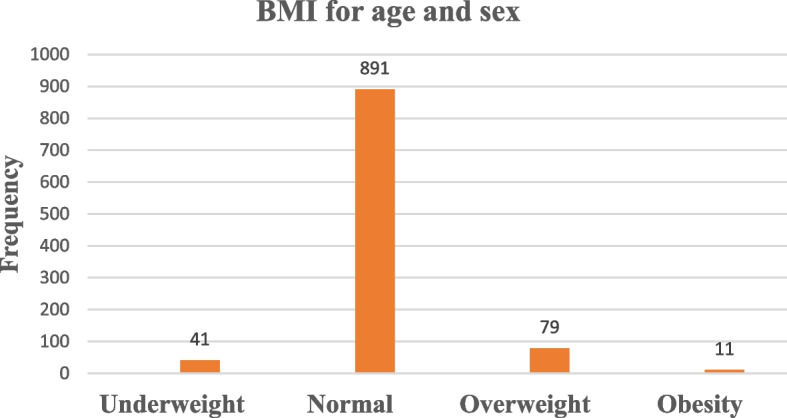
Body mass index for age and sex of public primary school children at Gamo zone, southern Ethiopia 2021

The median weight and mean height of respondents were 42.6 ± (IQR (interquartile range); 35.5-50.23) kg, and 1.5 ± 0.087 m respectively. Regarding body mass index, among all respondents, the majority of 891(87.2%) were normal and 79 (7.73%) were overweight (Fig. 3). Body mass index for age and sex of public primary school children at Gamo zone, southern Ethiopia 2021.

### Footwear related characteristics

The mean left and right foot sizes of the participants were 22.6 ± 1.349 cm and 22.67 ± 1.35 cm respectively. Of those 1022 children, 919 (89.92%) were found to wear shoes, while the remaining 103 (10.08%) did not wear shoes. Among those who wore shoes, 814 (88.57%) usually wore sandals. The majority of children 894 (97.28%) had started wearing shoes at an early age (Table 2).

**Table 2:** Foot related characteristics of primary school children in the Gamo zone, southern Ethiopia, 2021

Characteristics	Categories	Frequency (N)	Percent (%)
Do you wear shoes?	Yes	919	89.92
	No	103	10.08
Type of shoes you wear usually	Sandal	814	88.57
	Closed-toe shoe	105	11.43
At what age did you start wearing the shoe?	< 10 years	894	97.28
	≥ 10 years	25	2.72

### Physical activity and foot pain

Concerning physical activity, 927 (90.70%) of the study participants had sufficient physical activity, and the remaining 95 (9.30%) had insufficient physical activity. Regarding foot pain, 170 (16.63%) children had a history of foot pain in the past six months before data collection while 852 (83.37%) did not. Among children who had foot pain, 90 (52.9%) occasionally experienced pain while 43 (23.3%), and 37(21.7) frequently and rarely experienced foot pain, respectively. Of those who experienced foot pain, 123 (72.4%) participants reported that playing provoked foot pain while the remaining 30(17.6%) and 17(10%) reported walking and night (rest), respectively.

### The magnitude of flat foot

The overall magnitude of flat foot was 105 ((10.27%) 95% CI (8.5-12)) of which 20 (19.05%) were ridged and (80.95%) were flexible types of flat foot. Among 105 children with flat foot, 36 (34.3%) were symptomatic, and 69 (65.7%) were asymptomatic. Twenty-six (24.76%) were on the left leg, 35 (33.3%) were on the right and 44 (41.9%) were on both sides of the legs. The median ± (IQR) width of the narrowest part of the midfoot was 3.3 ± (2.5-4), and the width of the widest part of the hindfoot was 5.3 ± (5-5.7). The median ± (IQR) Staheli’s Plantar Arch Index was 0.6 ± (0.5-0.73).

### Factors associated with flat foot

Bivariable binary logistic regression was performed to assess the association of each independent variable with the outcome variable. All variables that were significantly associated, and had a *P*-value of less than 25% in bivariable analyses were entered into a multivariable logistic regression model using a backward variable selection method. These included altitude, residence, sex, wearing shoes, physical activity, foot pain, body mass index, and type of footwear. Among these, altitude, sex, residence, physical activity, foot pain, body mass index, and type of footwear were found to be independent factors associated with flat foot in multivariable logistic regression analysis (Table 2).

Male children were two times more likely to have flat foot than females (AOR = 2; 95% CI: 1.22-3.30. Children living in highland, and midland altitudes had 8.83, and 3.32 times higher likelihoods of flat foot than children living in lowland altitudes (AOR = 8.83; 95% CI: 5 4.64-16.79), and (AOR = 3.32; 95%CI: 1.75-6.29), respectively. Children residing in urban areas were 2.42 (AOR: 2.42; 95% CI: 1.15-5.09) more likely to have flat foot than rural residents. Children with insufficient physical activity were 8.78 times (AOR: 8.78; 9 95% CI: 4.42-12.3) more independently associated with flat foot than those with sufficient physical activity. On the other hand, wearing closed-toe shoes was 2.33 times (AOR: 2.33; 95% CI: 11 1.27-4.28) independently associated with flat foot than wearing sandals. Children having foot pain were 3.52 times (AOR: 3.52; 95% CI: 2.08-6.27) more likely to have flat foot than those with no foot pain. Being obese and overweight was 6.30 times (AOR: 6.30; 95% CI: 3.31- 11.9) more associated with flat foot than being underweight (Table 3).

**Table 3:** Bivariable and multivariable logistic regression analysis on factors associated with pes planus among public primary school children in Gamo Zone, Southern Ethiopia 2021

Variables	Pes planus		COR (95% CI)	AOR (95% CI)	*P*-values
	Yes (%)	No (%)			
Altitude					
Low	32 (5.86)	517 (94.14)	1		
Mid	36 (12.12)	261 (87.8)	2.21 (1.3- 3.6)	3.32 (1.75–6.29)	0.001*
High	37 (20.67)	142 (79.3)	4.2 (2.5- 6.95)	8.83 (4.64–16.79)	< 0.001*
Residence					
Rural	12 (5.2)	219 (94.81)	1		
Urban	93 (11.76)	698 (88.24)	2.43 (1.3–4.52)	2.42 (1.15–5.09)	0.019*
Age (in years)					
Early (11–14)	70 (10.17)	618 (89.83)	0.967 (0.63–1.48)		
Late (15–18)	35 (10.48)	299 (89.52)	1		
Sex					
Female	48 (8.45)	520 (91.55)	1		
Male	57 (12.56)	397 (87.44)	1.56 (1.03–2.3)	2.01 (1.22–3.30)	0.006*
Physical Activity					
Insufficient	25 (26.32)	70 (73.68)	3.78 (2.26–6.30)	8.78 (4.42–12.3)	< 0.001*
Sufficient	80 (8.63)	847 (91.37)	1		
BMI (kg/m^2^)					
Normal	77 ( 8.66)	812 (91.34)	1	0.33 (0.072- 1.56)	0.166
Underweight	3 (6.82)	41 ( 93.2)	0.77 (0.23–2.54)	6.30 (3.31- 11.9)	0.001*
Obese and overweight	25 ( 28.1)	64 (71.9)	4.12 (2.45–6.9)		
Foot pain					
Yes	36 (21.18)	134 (78.82)	3.04 (1.95–4.74)	3.52 (2.08–6.27)	< 0.001*
No	69 (8.10)	783 (91.90)	1		
Wear shoes					
Yes	99 (10.77)	820 (89.23)	1.95 (0.83- 4.56)	0.08 (0.003–1.92)	0.120
No	6 (5.83)	97 (94.17)	1		
Type of foot wear					
Closed-toe shoe	24 (30.77)	54 (69.23)	4.96 (2.89- 8.5)	2.33 (1.27–4.28)	< 0.001*
Sandal	71 (8.22)	793 (91.78)	1		
Age starting to wear shoes					
Early	92 (10.29)	802 (89.71)	1.66 (0.56- 4.94)		
Late	4 (16.00)	21 (84.00)	1		

*COR* crude odds ratio, *AOR* adjusted odds ratio, *1* reference category, *CI* confidence interval

^*^statistically significant variable at *p* < 0.05

### Correlation of SPAI with height and foot size of children

There was no significant correlation between SPAI and the height of the children (*r* = -0.0543, *p* = 0.0827). SPAI and size of both right and left foot were not significantly correlated (*r* = 0.0468 *p* = 0.1349, *r* = 0.0242 *p* = 0.4404).

### Comparison of male and female sex on SPAI

An independent sample t-test was conducted to see the mean difference in SPAI between sexes. The SPAI for males was 0.61 ± 0.24 and for females was 0.67 ± 0.37, which was statistically significant. (*P* = 0.01).

## Discussion

This study set out with the aim of assessing the magnitude and factors associated with a flat foot in Gamo Zone, Southern Ethiopia. The findings of this study revealed that the overall magnitude of flat foot was 105 (10.27%) 95% CI: 8.5-12). Sex, living altitude, residency, physical activity, type of footwear, BMI, and foot pain were statistically associated with flat foot.

This finding, the magnitude of flat foot was comparable to the study previously done in Ethiopia [17]. However, this finding was lower than a report from another study conducted in Ethiopia [13], Nigeria [14], India [3], and Saudi Arabia [23] 17.6%, 22.4%,16%, and 29.5% respectively. This variation might be due to the differences in the study area, sample size, socio-economic status, and differences in assessment tools, race, and heredity. For instance, the study conducted in Ethiopia encompasses only highland areas. The Indian study was conducted on only 50 participants and a higher percentage magnitude of flat foot in Saudi Arabia might be due to differences in socioeconomic status from the Ethiopian population.

The magnitude of flat foot was found to be highest among children from high altitude, followed by children from midland, and least in children from the lowland. Living in the highland areas increases the odds of having flat foot 8.83 times compared to those from the lowland. This was supported by a study done in Colombia [24]. This might be associated with the higher prevalence of vitamin D deficiency in children living in highland areas [25]. Vitamin D insufficiency occurs in 4% of cases every 100 m above sea level [25]. Vitamin D is vital for children's normal development of bone including the arch of the foot [26].

The present study revealed that the sex of participants was significantly associated with flat foot. Being male increases the odds of having a flat foot by two times. This result was supported by other studies performed in Nigeria [14], Austria [15], Indonesia, and Ethiopia [13]. The higher proportion of flat foot among male children could be explained by the greater rear foot valgus and retarded development of the rear foot in boys compared with girls [15]. Children with insufficient physical activity were found to have higher odds of pes planus as compared to their counterparts. Children with insufficient physical activity were 8.78 times more likely to have flat foot. This finding was consistent with other studies done in Pakistan and Ethiopia [12, 14]. This might be due to sufficient physical activity being very important for children’s muscular fitness and normal bone growth [23]. The results of this study showed that residence was significantly associated with flat foot. Children who live in urban settings were 2.42 times more likely to have flat foot than rural dwellers. This finding was in agreement with another study done in Ethiopia, which found that residents in urban areas had a higher likelihood of flat foot as compared to rural residents [22]. This might be related to the lifestyle difference between rural and urban residents. The present study revealed that the type of footwear was significantly associated with the magnitude of flat foot. This indicated that children who wear usually closed-toe shoes had a 2.33 times higher likelihood of flat foot than those who wore sandals. This finding was in agreement with studies performed in Nigeria and Ethiopia [5, 22]. A possible explanation might be that closed-toe shoes will limit medial arch development due to their tightness and rigidity compared to sandals. Body mass index status was significantly associated with the magnitude of flat foot. Being obese and overweight was found to increase the odds of flat foot by 6.3 fold. This finding was supported by studies done in Indonesia [27], Austria [15], and Ethiopia [13]. This might be due to the fact that abnormal nutritional status can affect normal body development, including medial arch development. Obese and overweight children are less involved in physical activities which in turn affect medial arch development. Being overweight and obese can increase the pressure due to excessive weight that is applied to the longitudinal arch, ligaments, and soft tissues of the foot, during stance and walking [27]. Foot pain was found to increase the odds of having a flat foot. Those children who had foot pain were 3.52 times more likely to have flat foot. This finding has been supported by a study performed in Gondar in 2020 [12]. This could be due to the pressure that lies up on misaligned feet.

In the current study, the results showed that there was no significant association between flat foot and age. This finding was supported by other studies conducted in Ethiopia [17], Nigeria [28], Indonesia [11], and Iran [29]. In contrast, pes plans were found to have a significant association with age, according to the findings from studies conducted in Nigeria [18], Saudi Arabia [23], and Austria [15]. The difference might be due to the skeletal immaturity of study participants in those studies. This is because the ossification of the foot bones may not be complete till the end of the first decade [30]. As children are younger they have more risk of having developmental (physiologic) flat foot. Age is a principal predictor for this type of flat foot but not for pathologic flat foot [22].

There are some limitations in this study. First, this study is a cross-sectional study which has the difficulty of clearly defining causative links between the factors studied and the outcome of interest. Second, the current study was institution-based and did not assess some important factors like family history and economic status.

## Conclusion

One in every ten children had a flat foot. Altitude, residence, sex, physical activity, foot pain, body mass index, and type of footwear were found to be factors statistically associated with flat foot. According to the findings of this study, it is recommended for children to wear sandals more frequently, have sufficient physical activity, and practice healthy eating habits. It is recommended that health care providers consider flat foot for children with foot pain of unknown origin. Possible solutions include routine mandatory flatfoot screening and monitoring for students. Further longitudinal research needs to be conducted to obtain an in-depth understanding of the risk factors for flat foot.

## Data Availability

The data sets used in this study for analysis and other information are available currently in the hands of the corresponding author and principal investigator. Therefore, it is possible to get with reasonable request.

## Abbreviations

AOR: Adjusted odds ratio
COR: Crude odds ratio
CI: Confidence interval
IQR: Interquartile range
MLA: Medial longitudinal arch
LLA: Lateral longitudinal arch
PI: Plantar index
SPAI: Staheli’s plantar arch index
VIF: Variance inflation factor
